# The Coexistence of Occult (Latent) Thyroid Cancer and Multinodular Goiter After Total Thyroidectomy: A Retrospective Study

**DOI:** 10.7759/cureus.62386

**Published:** 2024-06-14

**Authors:** Athanasios Permekerlis, Christos Tepelidis, Eirini Gemousakaki, Panagiotis Fotiadis

**Affiliations:** 1 Second Surgical Department, 424 General Military Hospital, Thessaloniki, GRC

**Keywords:** hashimoto’s thyroiditis, retrospective research, histologic examination, sub-total thyroidectomy, multinodular goiter

## Abstract

Introduction

Multinodular goiter (MNG) is a common thyroid disorder characterized by the presence of multiple nodules within the thyroid gland. While most cases of MNG are benign, there is a risk of malignancy, particularly in nodules with certain features. The coexistence of occult (latent) thyroid cancer within MNG presents diagnostic and management challenges, underscoring the need for comprehensive investigation and treatment strategies.

Objective

The objective of this retrospective study is to investigate the prevalence of occult thyroid carcinoma in non-toxic MNG following total thyroidectomy.

Materials and methods

The study population consisted of 412 patients who underwent total thyroidectomy between 2004 and 2022 at the Second Surgical Department of the 424 General Military Hospital of Education in Thessaloniki. Data collection included patients' demographic characteristics, surgical indications for thyroidectomy, and histopathological examination findings. Initial data were available for all 412 patients, while sufficient information was present for 319 individuals, with a subset of 271 undergoing total thyroidectomy due to non-toxic MNG. Out of the aforementioned group, 253 cases were histologically confirmed as MNG. Subsequently, a statistical analysis was conducted concerning age, gender, the association of MNG with malignancy, and other thyroid disorders.

Results

Out of the total 412 thyroidectomies performed, 271 patients remained for statistical analysis and study. Among them, 253 patients had histologically confirmed MNG. Among the histological findings, 38 cases (14.02%) were identified with occult carcinoma within MNG. The predominant histological type was papillary thyroid carcinoma (PTC), comprising 93.3% of cases. Additionally, 18 patients (6.64%) were diagnosed with MNG, Hashimoto's thyroiditis (HT), and malignancy concurrently.

Conclusions

The coexistence of occult thyroid carcinoma within MNG underscores the importance of vigilant evaluation and management strategies in patients undergoing total thyroidectomy. These findings emphasize the need for comprehensive preoperative assessment and postoperative surveillance to detect and address occult thyroid cancer, thereby optimizing patient care and outcomes.

## Introduction

The term multinodular goiter (MNG) is used to describe the enlargement of the thyroid gland. Various criteria have been utilized to define it, including ultrasonographic criteria (thyroid volume increase by two times the normal volume), and clinical criteria (palpation of a large thyroid gland with nodular consistency) [1,2]. Generally, according to certain studies, it is defined as ~18-19 mL for the female population and ~25 mL for males [3].

Depending on the morphological characteristics of the gland, it is distinguished as diffuse (diffuse enlargement of the thyroid size) and nodular (when one or more nodules are found, with a different shape or function from the rest of the thyroid parenchyma) [4]. Subsequently, if there is one nodule, it is defined as a solitary thyroid nodule, whereas if there are multiple nodules, it is defined as MNG.

Furthermore, MNG can be classified as sporadic or endemic based on epidemiological criteria. It is defined as sporadic if the prevalence is <10%, whereas it is considered endemic if it is >10% in a geographic area [5].

Finally, MNG is differentiated into toxic (autonomous production and secretion of thyroid hormones) or non-toxic (absence of functional activity) depending on thyroid function. Typically, patients with non-toxic MNG are euthyroid, but a significant portion of patients suffer from hypothyroidism.

Thyroid nodules are a common clinical finding in areas with iodine deficiency, affecting approximately 10% of the general population. They are more prevalent in females. Around 4% of patients present clinically evident nodules, while with the use of ultrasound, this incidence reaches up to 50% [6,7]. Predisposing factors for malignancy seem to include male gender, a history of head and neck radiation, and age (under 30 years, over 60 years). Recent studies indicate a similar likelihood of malignancy between MNG and solitary nodules, contrary to earlier theories that strongly associated solitary nodules with malignancy.

Surgical intervention for non-toxic MNG is warranted in several scenarios. These include cases where the goiter exhibits significant size or shows enlargement during follow-up assessments. Additionally, surgical consideration arises when patients manifest compression symptoms like dyspnea, Pemberton’s sign, dysphagia, or related findings. The presence of a substernal goiter, identification of a malignant or suspected malignant nodule, or concerns related to visual impairment also indicate the need for surgery. Furthermore, a non-diagnostic result from fine needle aspiration biopsy (FNAb) can prompt surgical intervention in managing non-toxic MNG.

This retrospective study investigates the presence of occult thyroid carcinoma in non-toxic MNG.

## Materials and methods

The study population consisted of 412 patients who underwent total thyroidectomy between 2004 and 2022 at the Second Surgical Department of the 424 General Military Hospital of Education in Thessaloniki. Data collection included patients' demographic characteristics, surgical indications for thyroidectomy, and histopathological examination findings. Initial data were available for all 412 patients, while sufficient information was present for 319 individuals, with a subset of 271 undergoing total thyroidectomy due to non-toxic MNG. Out of the aforementioned group, 253 cases were histologically confirmed as MNG. Subsequently, a statistical analysis was conducted concerning age, gender, the association of MNG with malignancy, and other thyroid disorders (Table 1).

**Table 1:** Study Population and Inclusion Criteria MNG, multinodular goiter

Study Population	
Total Patients	412
Sufficient Data	319
Surgical Indication: MNG	271
Histological Confirmed MNG	253

## Results

Out of the total 412 thyroidectomies performed at our clinic from 2004 to 2022, based on the exclusion criteria (sufficient data, indication for total thyroidectomy), 271 patients remained for statistical analysis and study. Among them, 253 patients had histologically confirmed MNG. The remaining 18 patients, operated with the preoperative indication of MNG, did not confirm histologically and were found to suffer from other thyroid disorders (malignancy, Hashimoto's thyroiditis (HT)). The final criterion for selecting the study population was the histologically confirmed coexistence of MNG and malignancy.

The age range of patients who underwent total thyroidectomy ranged from 15 to 79 years, with a mean age of 48.7 years. The gender distribution was predominantly female to male at 7:1. Among these patients, the primary clinical indications included an increase in thyroid gland size during follow-up, symptoms of compression, and non-diagnostic findings in the FNA.

Among the histological findings from the surgical specimens of the 271 patients, 253 cases were diagnosed with MNG (93.3%), 70 patients presented with HT (25.3%), and 54 patients were diagnosed with malignancy (19.9%). Subsequently, potential correlations between these conditions were investigated (Table 2).

**Table 2:** The Histological Distribution of the Patients MNG, multinodular goiter; HT, Hashimoto thyroiditis

The Histological Distribution of the Patients	
MNG	93.35%
HT	25.3%
Malignancy	19.9%
MNG - Malignancy	14.02%
MNG - HT	22.1%
MNG - HT - Malignancy	4.05%

Among the subgroup of patients with histologically confirmed MNG and malignancy, there were 38 cases (14.02%). Their age ranged from 29 to 70 years, with a mean age of 49.8 years. The gender distribution was 4.5:1, predominantly skewed toward females. This percentage reflects the rate of occult carcinoma in MNG. The above percentage reflects the rate of occult carcinoma within MNG. In 37 cases (93.3%), the histological type was papillary thyroid carcinoma (PTC), while only one case presented as follicular (2.7%).

The total number of patients identified in the histological examination of the surgical specimens with MNG - HT - Malignancy was 18 (6.64%). The mean age was 50 years, with a female predominance of 4.5:1.

## Discussion

MNG is a common thyroid disorder characterized by the presence of multiple nodules within the thyroid gland. While most cases of MNG are benign, there is a risk of malignancy, particularly in nodules with certain features. Therefore, the management of MNG often involves surgical intervention to alleviate symptoms, confirm diagnosis, and address the risk of malignancy [8].

Epidemiology of multinodular goiter

MNG represents a significant portion of thyroid disorders worldwide, with varying prevalence rates across different populations. The incidence of MNG tends to increase with age, and it is more common in women than in men. Environmental factors such as iodine deficiency can also influence the prevalence of MNG in certain regions [1].

Multinodular goiter and the possibility of cancer

While most nodules in MNG are benign, a small percentage can harbor malignancy. The risk of cancer in MNG varies depending on factors such as nodule size, patient age, and the presence of suspicious features in imaging studies. According to a retrospective cohort study involving over 8,000 patients, the rate of completion of thyroidectomy for incidental thyroid cancer in MNG was found to be higher in cases treated with lobectomy compared to total thyroidectomy (2.15% vs. 0.1%, respectively). Furthermore, the recurrence rate in patients who underwent lobectomy was significantly higher, with a considerable proportion requiring reoperation [9].

Carcinoma of the thyroid: epidemiology and common types

Thyroid carcinoma encompasses a group of malignant tumors arising from the thyroid gland. The most common type of thyroid carcinoma is PTC, accounting for approximately 80-85% of all cases. Follicular thyroid carcinoma (FTC) is the second most prevalent type, comprising around 10-15% of cases. Other less common types include medullary thyroid carcinoma (MTC), accounting for about 3-5% of cases, and anaplastic thyroid carcinoma (ATC), which represents less than 2% of cases. The incidence of thyroid carcinoma has been increasing globally, with papillary carcinoma being the main contributor to this trend [10].

Solitary nodule of the thyroid and the risk of cancer

While solitary thyroid nodules are less common than MNGs, they also carry a risk of malignancy. The percentage of solitary nodules that are malignant varies depending on factors such as patient age, nodule size, and the presence of suspicious features in imaging studies. According to a retrospective cohort study, the rate of malignancy in solitary nodules can range from 5% to 15%, with larger nodules and older age being associated with a higher risk [11].

Risk factors of multinodular goiter for cancer

Several risk factors have been identified for the development of thyroid cancer in the setting of MNG. These include a history of radiation exposure, a family history of thyroid cancer, and certain genetic syndromes such as familial adenomatous polyposis (FAP) and Cowden syndrome. Additionally, the presence of specific ultrasound features such as microcalcifications, irregular margins, and increased vascularity can increase the suspicion of malignancy in nodules within an MNG [12].

Features of ultrasound-guided fine needle aspiration (FNA) and Bethesda score

Ultrasound-guided fine needle aspiration (FNA) is a valuable tool in the evaluation of thyroid nodules, including those within an MNG. FNA allows for the sampling of individual nodules within an MNG, helping to differentiate between benign and malignant lesions. The Bethesda System for Reporting Thyroid Cytopathology provides a standardized framework for interpreting FNA results, categorizing them into six diagnostic categories based on the risk of malignancy [13].

Fine needle aspiration and multinodular goiter

In patients with MNG, FNA can help determine the nature of individual nodules and guide further management. Nodules that are suspicious for malignancy or indeterminate on FNA may warrant surgical excision for definitive diagnosis and treatment. However, it is essential to consider the overall clinical context, including the patient's age, comorbidities, and preferences, when deciding on the appropriate management approach [14].

The most appropriate surgery for multinodular goiter

The optimal surgical approach for MNG depends on various factors, including the size and location of nodules, the presence of suspicious features on imaging, and the patient's overall health status. Total thyroidectomy is often recommended for bilateral disease, suspicion of malignancy, or symptomatic enlargement. However, in cases of unilateral disease without concerning features, thyroid lobectomy may be sufficient, preserving thyroid function and minimizing the risk of complications such as hypoparathyroidism and recurrent laryngeal nerve injury [15].

Surgical management of multinodular goiter: evidence and recommendations

Several studies have compared the outcomes of different surgical approaches for MNG, including total thyroidectomy, bilateral subtotal thyroidectomy (BST), and thyroid lobectomy. A retrospective cohort study involving over 8,000 patients found that the rate of completion of thyroidectomy for incidental thyroid cancer was higher in cases treated with BST compared to total thyroidectomy, with a higher recurrence rate observed in patients who underwent BST [10].

In a literature review evaluating studies published between 1987 and 2007, temporary hypoparathyroidism was found to be more common in patients undergoing TT compared to subtotal thyroidectomy, with no significant difference in permanent complication rates between the two groups. However, the recurrence rate was higher in patients who underwent subtotal thyroidectomy [16].

A prospective randomized study evaluating the 10-year follow-up results of different surgical techniques in the treatment of MNG found that total thyroidectomy was associated with a lower recurrence rate compared to both BST and the Dunhill procedure. Additionally, total thyroidectomy did not increase the total permanent complication rate and eliminated the risk of recurrence and reoperation, leading the investigators to recommend it as the preferred intervention for MNG [17].

## Conclusions

In conclusion, MNG is a common thyroid disorder that can carry a risk of malignancy, particularly in nodules with suspicious features. Ultrasound-guided fine needle aspiration plays a crucial role in the evaluation of thyroid nodules within an MNG, helping to guide further management decisions. Surgical intervention may be warranted in cases of suspicion of malignancy or symptomatic enlargement, with total thyroidectomy being recommended in certain scenarios to minimize the risk of recurrence and complications. Further research is needed to refine the management strategies for MNG and optimize patient outcomes.
